# Li-Doping-Induced Structural and Electronic Structure Modulation in MgTiO_3_ for an Electrochemical Energy Storage Supercapacitor Device

**DOI:** 10.1021/acsomega.6c00638

**Published:** 2026-07-01

**Authors:** Priyanka, Aditya Sharma, Bhavi Agrawal, Mayora Varshney, Shalendra Kumar, Hyun Joon Shin, Keun Hwa Chae, Jitendra Pal Singh, Jai Parkash

**Affiliations:** † Department of Sciences (Physics), 584797 Manav Rachna University, Faridabad, Haryana 121004, India; ‡ Department of Physics, 114800 University of Petroleum and Energy Studies, Dehradun, Uttarakhand 248007, India; § School of Applied & Life Sciences, UIT, & Division of Research & Innovation, 390541 Uttaranchal University, Dehradun, Uttarakhand 248007, India; ∥ Department of Physics, Chungbuk National University, Cheongju 28644, South Korea; ⊥ Advanced Analysis & Data Centre, Korea Institute of Science and Technology, Seoul 02792, South Korea

## Abstract

Correlation among the crystal structure, electronic structure, and electrochemical energy storage mechanism has been investigated for Li-doped MgTiO_3_ ceramics, prepared using the solid-state reaction method. Li doping leads to an increase in the occupied density of states in the band structure of MgTiO_3_, leading to a diminishing of the t_2g_/e_g_ peak intensities in the O K-edge and Ti L-edge XANES spectra. Though Li doping did not change the Ti^4+^ and Mg^2+^ oxidation states, upon increasing Li content, the Mg_2_TiO_4_ phase thrived. Under the three-electrode configuration, with KOH as the electrolyte, the MgTiO_3_, 5Li-MgTiO_3_, and 10Li-MgTiO_3_ samples have shown surface-plus-diffusion-based energy storage and offered high specific capacitances of 81.0 F/g, 190.5 F/g, and 309.0 F/g, respectively (at a scan rate of 5 mV/s). A Swagelok cell, a two-electrode-based symmetric supercapacitor device, has been investigated, which delivered an energy density of 46 Wh/kg at a power density of 5000 W/kg at a current density of 2.5A/g, retained greater than ∼71% capacity after 10,000 cycles, and powered an LED, indicating excellent practical energy storage performance.

## Graphical abstract

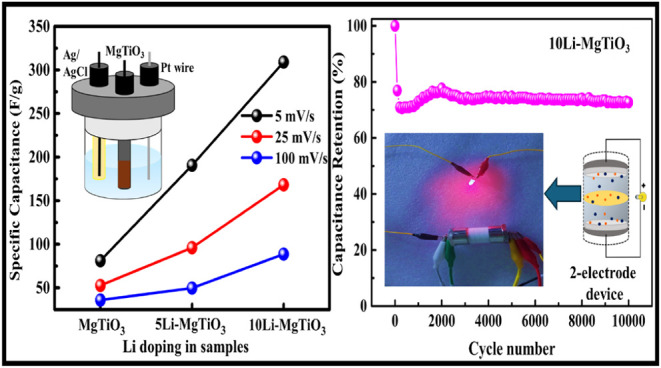

## Introduction

Supercapacitors are one of the solutions for energy storage technology among various types of energy storage devices like Li/Na/Zn ion batteries, fuel cells, etc.^1–7^ Supercapacitors offer high power density, rapid charging and discharging, long calendar life, and low manufacturing cost.^6,7^ One of the supercapacitor types is the electrochemical double-layer capacitors (EDLCs), developed by carbon-based materials, that store energy through electrostatic charge accumulation at the electrode–electrolyte interface. This kind of electrostatic charge storage mechanism of EDLCs leads to poor energy density, as it depends entirely on surface adsorption rather than bulk redox processes.^8^ Their low working voltage and limited surface area restrict their energy density. In contrast, pseudocapacitors overcome this limitation by enabling fast and reversible surface oxidation–reduction processes, which allow for higher energy density without compromising power density.^9^ For example, the specific capacitance of exfoliated graphene, GO, and rGO has been reported to be 14.8 F/g, 164.6 F/g, and 137.4 F/g, respectively.^9^ Similarly, conducting polymers have been reported to exhibit Faradaic-type supercapacitance behavior because of their good electrical conductivity and electrochemical characteristics. However, polymers experience poor cyclic performance because of shrinkage/expansion effects during charging and discharging cycles.^10^ Comparing carbonaceous materials , polymers, and other electrode materials, the metal oxide (MO)-based electrodes are interesting candidates because of the existence of various oxidation states in transition metal elements (e.g., Fe^2+^/Fe^3+^/Fe^4+^, Ti^2+^/Ti^4+^, etc.). The variable oxidation states of transition metal ions can offer reversible redox reaction-based electrochemical features in the cyclic voltammetry and support the pseudo capacitance-type charge storage mechanism.^11^ Many contemporary MOs have been reported to exhibit moderate specific capacitance values, such as pure CeO_2_ (91 F/g),^12^ pure ZnO (5.87 F/g),^13^ pure SnO_2_ (47 F/g),^14^ pure Bi_2_O_3_ (36 F/g),^15^ pure CaTiO_3_ (∼140 F/g),^16^ etc. The strategic doping of foreign elements into the MO host can enhance the specific capacitance, and doped MO’s have been reported to achieve elevated capacitance values, such as Ce-doped NiO (240 F/g),^17^ Mn-doped Co_3_O_4_ (221 F/g),^18^ Ni-doped Co_3_O_4_ (299 F/g),^19^ Cd-doped ZnO (311 F/g),^20^ and Zr-doped ZnO (375 F/g).^21^ Such improvements are attributed to induced defect formation, enhanced electronic conductivity, and greater redox activity in TMOs. Similar effects of dopant incorporation in oxide materials have also been reported in previous studies.^22,23^

To increase the specific capacitance of supercapacitor electrodes, recent research focuses on innovative materials and hybrid architectures.^24^ Likewise, the doping of alkali metal elements (Li, Na, and K) or the application of alkali metal-based oxide compounds is an advanced strategic step toward enhancing the specific capacitance of oxide compounds. The presence of alkali elements in the electrode material has shown improved electrochemical performance due to the addition of redox peaks from the plug-in and plug-out paths of alkali ions: TMO + A^+^ + e^–^ ↔ TMOA (A = alkali element).^25–29^ Therefore, in pursuit of high specific capacitance from the MO-based compounds, this research attempts to incorporate Li ions into the MgTiO_3_ oxide system. The choice of MgTiO_3_ was made as a test material based on its structural and dielectric properties.^30–33^ It obeys a rhombohedral ilmenite-type structure (trigonal symmetry; space group R3̅) in which Mg^2+^ and Ti^4+^ ions occupy octahedral sites in alternating layers along the *c*-axis, creating a highly ordered arrangement. The MgTiO_3_ shows low dielectric loss, excellent thermal stability with a coefficient of thermal expansion of 8–9 × 10^–6^/K, band gap energy of >3.0 eV, and electrical conductivity of the order of 10^–14^ S/cm.

The synthesis of monophase MgTiO_3_ has been challenging using various synthesis methods. Sol–gel-prepared MgTiO_3_ has been reported to exhibit secondary phases of MgTi_2_O_5_ and/or Mg_2_TiO_4_ when prepared at 600 °C.^30^ Autoignited combustion method-prepared MgTiO_3_ nanoparticles exhibited MgTi_2_O_5_ at 800 °C.^34^ A coprecipitation method was applied to synthesize MgTiO_3_ ceramic powders, but a secondary phase of Mg_2_TiO_4_ was observed at 1000 °C annealing temperatures.^35^ Sono-chemically prepared MgTiO_3_ nanoparticles have shown secondary phases of TiO_2_.^36^ Despite the several advantages of chemical route-based synthesis methods, there is limited applicability of method-originated materials in the ceramic industry. Therefore, it is imperative to prepare ceramic materials using the solid-state reaction method. Though many attempts have been made to prepare single-phase MgTiO_3_ ceramics, Mg_2_TiO_4_ and/or MgTi_2_O_5_ phases were always present in the solid-state reaction method-synthesized products.^37–39^ The aliovalent ion (e.g., Li^+^) doping can modulate secondary phase formation and their concentrations, create oxygen defects, improve ionic mobility, reduce the band gap energy, and enhance the overall ionic conductivity of MgTiO_3_.^40,41^ Therefore, the incorporation of Li has been strategically reported to improve the electrochemical performance of MgTiO_3_ ceramics.^33^ In this report, two different Li concentrations have been applied (5 and 10 wt %), compared to the Mg content in the MgTiO_3_ compound. The effect of Li doping on the structural, electronic structure, and electrochemical traits has been investigated in pursuit of an aqueous symmetric supercapacitor device. The Li-doped MgTiO_3_ has offered high specific capacitance and demonstrated Swagelok cell device functionality to glow a red LED.

## Materials and Methods

### Material Synthesis

For the synthesis of the material, reagents were used without further purification and were procured from Sigma-Aldrich, having a purity greater than 99.9%. For the preparation of all samples, a conventional solid-state reaction method was applied. This method has been reported for preparing other pure and doped oxide compounds to diversify properties.^42–45^ A stoichiometric mixture of MgO and TiO_2_ was ground for 2 h using an agate mortar and pestle and calcined at 500 °C for 2 h. After cooling to room temperature, the sample was further ground for 2 h and sintered at 1200 °C for 12 h. For the preparation of the Li-doped materials, 5Li-MgTiO_3_ and 10Li-MgTiO_3_, stoichiometric proportions of LiOH were introduced along with MgO and TiO_2_ precursors. The mixed powders were subjected to grinding for 2 h using an agate mortar and pestle. Then, the above-mentioned processes of annealing at 500 °C and sintering at 1200 °C were applied. The overall synthesis procedure of pure and Li-doped MgTiO_3_ samples is illustrated in Scheme S1 (ESI File).

### Characterization Details

X-ray diffraction (XRD) measurements were carried out using a Rigaku-Miniflex 600 X-ray diffractometer (λ = 1.5418 Å). Fourier-transformed infrared spectroscopy (FTIR) measurements were performed using the Shimadzu IRSpirit-T machine with attenuated total reflectance (ATR) mode. Cyclic voltammetry (CV), galvanostatic charging–discharging (GCD), and electrochemical impedance spectroscopy (EIS) measurements were performed (for three-electrode and two-electrode configurations) using the Corrtest CS2350 M electrochemical workstation. Scanning electron microscopy (SEM) and energy-dispersive X-ray spectroscopy (EDS) measurements were performed using the ZEISS EVO-15 machine. XANES spectra of the O K-edge and Ti L-edge were collected in total electron yield (TEY) mode at the soft X-ray beamline, 10D-XAS-KIST, at Pohang Accelerator Laboratory (PAL), South Korea.

### Electrode Preparation and Electrochemical Characterization

For all electrochemical characterizations, a 1 M aqueous solution of KOH was utilized as an electrolyte. For developing the working electrode, the previously stabilized drop-casting method is applied^25,27,46,47^ as shown in Scheme S2 (ESI file). The experimental details (for 2-electrode and 3-electrode configurations) and mass loading values are provided in the ESI file and Table S2.

## Results and Discussion

### X-ray Diffraction Study

Figure 1a shows the Rietveld-refined XRD patterns of MgTiO_3_, 5Li-MgTiO_3_, and 10Li-MgTiO_3_ samples. It is noticeable that all three samples exhibit intense XRD peaks, indicating convincing crystalline compound fabrication under the given synthesis protocols. The XRD patterns of pure MgTiO_3_ were tallied with the ICDD database and matched with JCPDS card number 06-0494, corresponding to an ilmenite-type crystal structure (trigonal crystal system; space group R3̅). The XRD peaks at 19.25°, 21.43°, 24.16°, 33.04°, 35.62°, 40.76°, 53.71°, and 57.02° correspond to the (003), (101), (012), (104), (110), (113), (107), (116), and (018) planes, respectively. The diffraction peaks of pure MgTiO_3_ are also well-matched with previous reports^30–32^ and confirm the synthesis of the crystalline MgTiO_3_ phase in the present study. Some low-intensity XRD peaks are also present in the XRD data of pure and Li-doped MgTiO_3_ samples (marked by *). These low-intensity peaks are matched with the crystallographic phase of Mg_2_TiO_4_.^30,31^ For better illustration of the XRD peaks of Mg_2_TiO_4_ phase, the intensity (*y*-axis) is plotted on a log scale, and the XRD patterns are presented in Figure S1 (ESI file). In many previous reports, both the secondary phases (Mg_2_TiO_4_ and MgTi_2_O_5_) were grown at annealing treatments of ≥1000 °C.^30–32^ It is apparent from Figure 1 and Figure S1 that the XRD peak intensity of the Mg_2_TiO_4_ phase is negligible in the pure MgTiO_3_ sample and improves upon Li doping. This suggests the growth of an Mg-rich phase (i.e., Mg_2_TiO_4_) upon Li doping. The quantitative phase analysis, using Rietveld refinements, suggested ∼2% Mg_2_TiO_4_ phase in the undoped sample, which increases to ∼15% for the 10Li-MgTiO_3_ sample. The effect of Li doping on the lattice parameters of MgTiO_3_ was also evaluated using Rietveld refinement. The lattice parameters and Rietveld-refined parameters are presented in Table S1 (ESI file). For all the fittings, slightly higher values of χ^2^ and R factors were observed . This could be due to the high background and contribution from Cu Kβ radiation in the XRD data collected from the low-power-based tabletop XRD machine (tube voltage 40 kV, tube current 15 mA, which does not use Ni filters for Cu Kβ removal). It is apparent that the lattice parameters (a, b, c, and unit cell volume) of MgTiO_3_ are less affected at low Li concentrations but slightly decrease for the 10Li-MgTiO_3_ sample. This could be due to the doping of Li^+^ ions into the lattice of MgTiO_3_. Though Mg atoms are slightly larger (atomic radius is 160 pm) than Li ions (atomic radius is 152 pm), the ionic radius of Li^+^ ions (76 pm) is larger than that of Mg^2+^ ions (66 pm). Therefore, the substitution of Mg^2+^ with Li^+^ could enlarge the cell volume. However, a slight decrease in the unit cell volume indicates the occupation of other lattice sites by Li (possibly interstitial or grain boundaries) in the MgTiO_3_ lattice. The crystallite size was evaluated using the Scherrer relation: *D* = 0.9.*λ*/*β*cos*θ*. Here, *D* represents the crystallite size of the sample, β indicates the fwhm of the XRD peak, λ denotes the X-ray’s wavelength (1.5405 Å), and θ represents the angle of the XRD peak. The estimated average crystallite sizes are 74, 72, and 67 nm for MgTiO_3_, 5Li-MgTiO_3_, and 10Li-MgTiO_3_ samples, respectively. A slightly smaller crystallite size in Li-doped samples indicates the occupation of Li at grain boundaries, where it may hinder crystallite growth.

**Figure 1:**
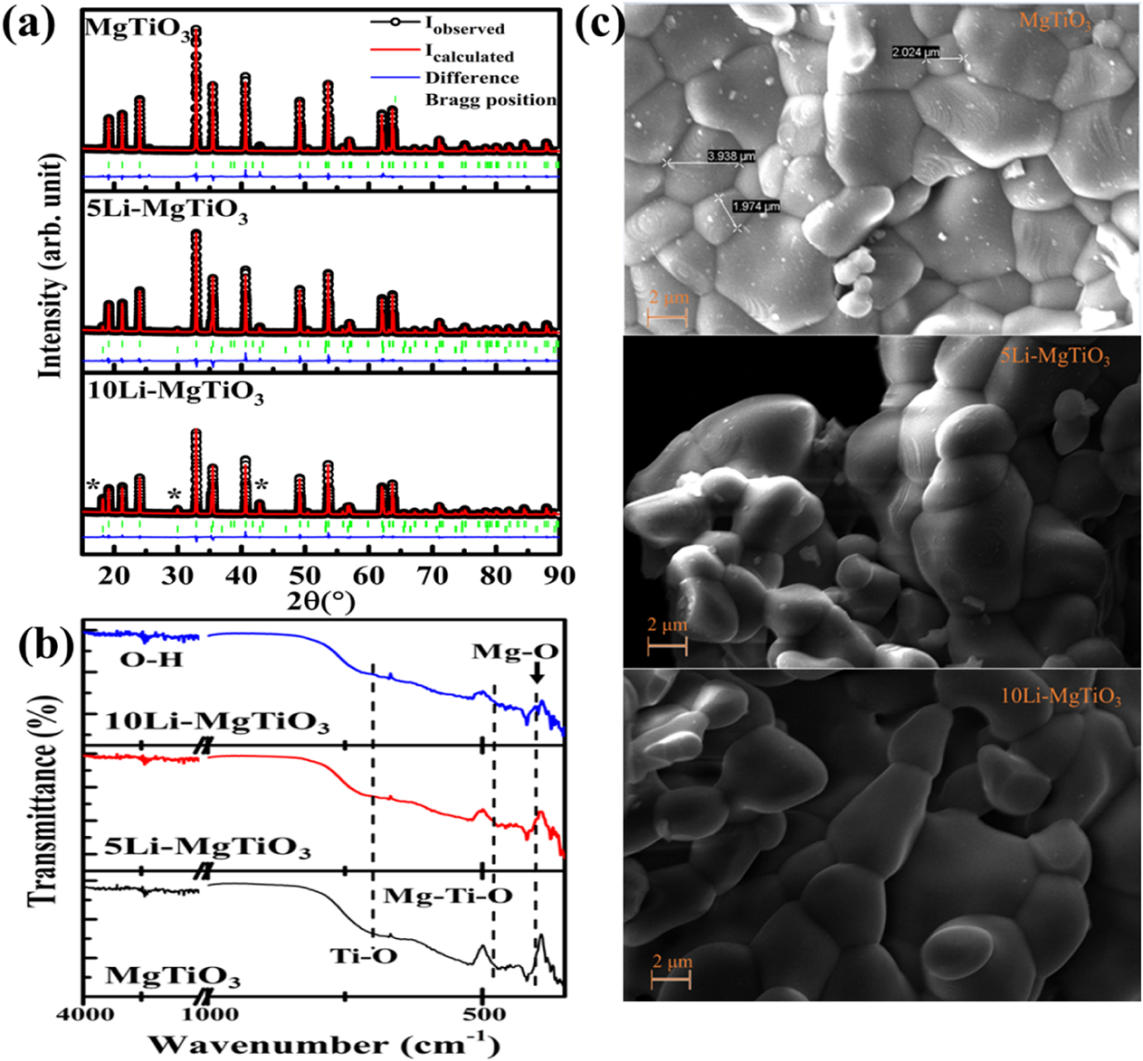
(a) Refined XRD patterns, where (*) shows XRD peaks from the Mg_2_TiO_4_ phase, (b) FTIR spectra, and (c) SEM images of MgTiO_3_, 5Li-MgTiO_3_, and 10Li-MgTiO_3_.

### FTIR Study

The FTIR spectrum was analyzed to determine the presence of functional groups and is presented in Figure 1 (b). The FTIR spectrum shows a distinctive band at 404 cm^–1,^ which is attributed to the presence of Mg–O stretching vibration. The band at 457 cm^–1^ could be due to the Mg–Ti–O vibrations. Also, the absorption bands at around 672 cm^–1^ may be attributed to the Ti–O vibrations.^48^ The broad band between 3820 and 3610 cm^–1^ is ascribed to the bending vibrations of O–H due to moisture on the surface of particles.^48^

### SEM–EDS Study

Figure 1c shows the SEM images of the MgTiO_3_, 5Li-MgTiO_3_, and 10Li-MgTiO_3_ samples. It is noticeable that a compact morphology of larger-sized particles is formed for all samples. A histogram, for estimating the size of particles, is presented in Figure 2a–c. Histogram shows that all the samples obey a size distribution within the range of 2–6 μm. The formation of large-sized particles is anticipated because of the high-temperature annealing-induced grain growth through Ostwald ripening, where the smaller-sized grains collapse to grow into larger-sized particles.^43,44^ Large-sized particle formation has been reported in other studies for high-temperature sintered samples.^43,44^ The EDS spectra are presented inFigure [Fig fig2]d-f and convey the existence of the constituent elements Mg, Ti, and O in each sample. The existence of an Au-related peak is due to the Au coating applied during the SEM-EDS measurements.

**Figure 2:**
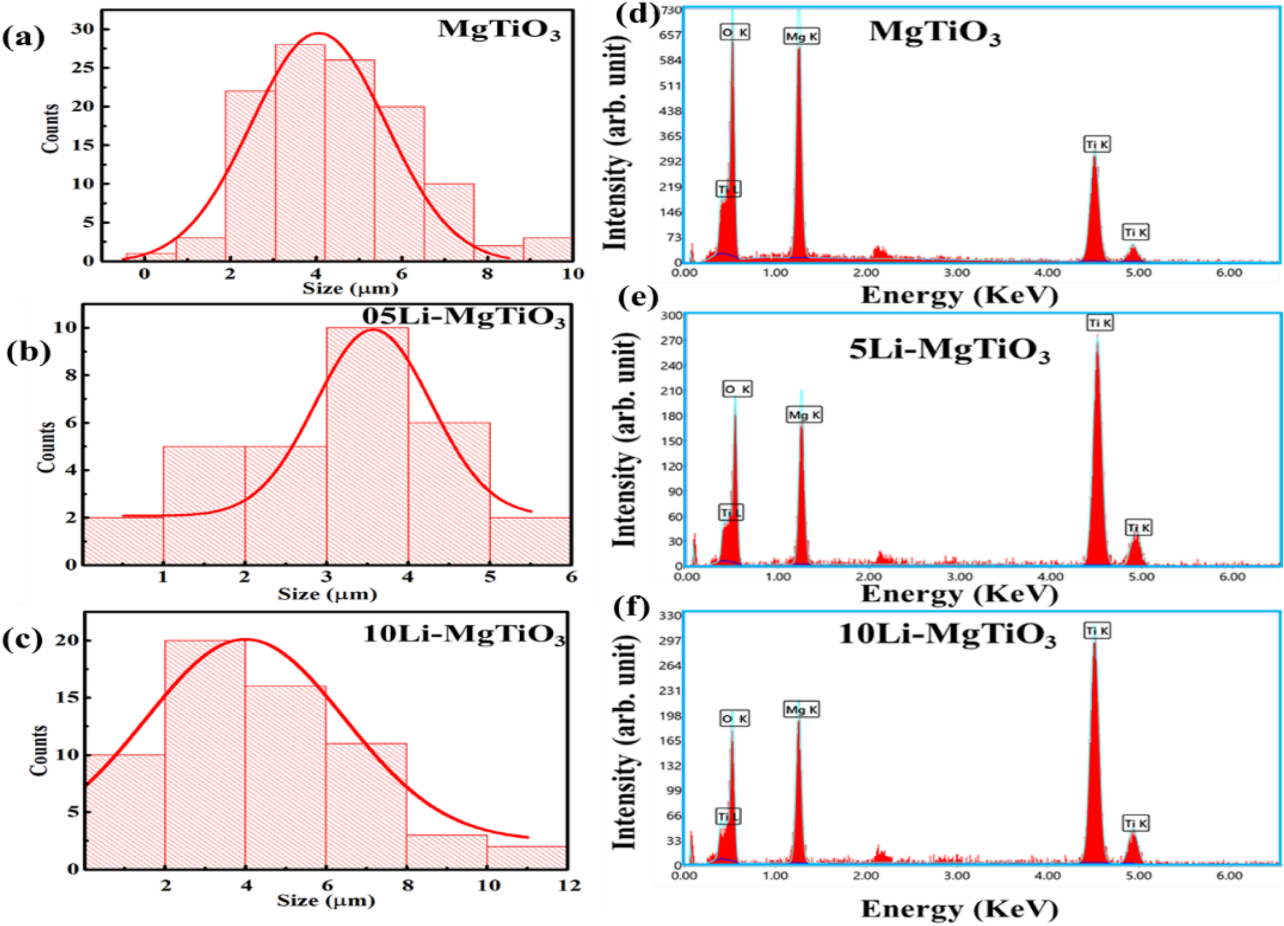
(a–c) Particle size distribution; (d–f) EDS spectra of MgTiO_3_, 5Li-MgTiO_3_, and 10Li-MgTiO_3_.

### XANES Investigation

Figure 3 shows the XANES spectra of (a) O K-edge, (b) Ti L-edge, and (c) Mg K-edge. For comparison of O K-edge and Ti K-edge XANES spectra, the TiO_2_ reference sample was also investigated under the same experimental conditions. Our XRD results confirmed that as-prepared MgTiO_3_ obeys a trigonal crystal structure, which consists of octahedrally coordinated Mg^2+^ and Ti^4+^ ions with six surrounding oxygen atoms. In this structural arrangement, the top of the valence band of MgTiO_3_ mainly involves the O-2p orbitals. The Ti-3d orbitals predominantly construct the bottom of the conduction band, with a small contribution from the hybridized O-2p and Mg-3s orbitals.^49^ The O K-edge XANES involves excitation of O 1s core electrons to an unoccupied p-symmetric final state, which may have hybridized with the metal d-states.^46^ The O K-edge XANES of MgTiO_3_ samples can be divided into two regions: the low energy (530–537 eV) and high energy (538–555 eV). The low-energy spectral features arise due to oxygen ligand field-induced crystal field splitting of the Ti 3d orbitals into low-energy t_2g_ and high-energy e_g_ levels. The t_2g_ and e_g_ features are magnified and presented on the left side of Figure 3a panel. It is noticeable from the O K-edge XANES that the t_2g_ and e_g_ peak positions are nearly the same for pure and Li-doped samples, but a decrease in the peak intensity is observed upon Li doping (e.g., t_2g_ peak). The decrease in the intensity of spectral features may be associated with (i) formation of filled t_2g_ and e_g_ states, (ii) reduced hybridization between O 2p and Ti 3d, and (iii) formation of oxygen vacancies.^46^ In pristine MgTiO_3_, the Ti^4+^ ions possess a 3d^0^ electronic configuration (i.e., t_2g_ or e_g_ states are largely unoccupied), leading to significant peak intensity in the O K-edge XANES of pure MgTiO_3_. Li^+^ doping in MgTiO_3_ may substitute Ti^4+^ ions, leading to a charge imbalance. This charge imbalance may be overcome via the formation of partial Ti^3+^ ions with a 3d^1^ electronic configuration, resulting in partially occupied t_2g_ /e_g_ states and offering a decreased peak intensity of O K-edge features. Likewise, the size and charge imbalance with Li in MgTiO_3_ can cause distortion in Ti–O bonds, leading to weakening the hybridization of O 2p and Ti 3d orbitals (i.e., π-type interactions associated with _*t*2g_ levels). The weakened hybridization between O 2p and Ti 3d orbitals may also suppress the O K-edge peak intensity. Lastly, to compensate for the charge, oxygen ion vacancies can also be formed. Such oxygen ion vacancies can disrupt the Ti–O linkages in MgTiO_3_ and, consequently, lower the peak intensity of the O K-edge XANES spectra.

**Figure 3:**
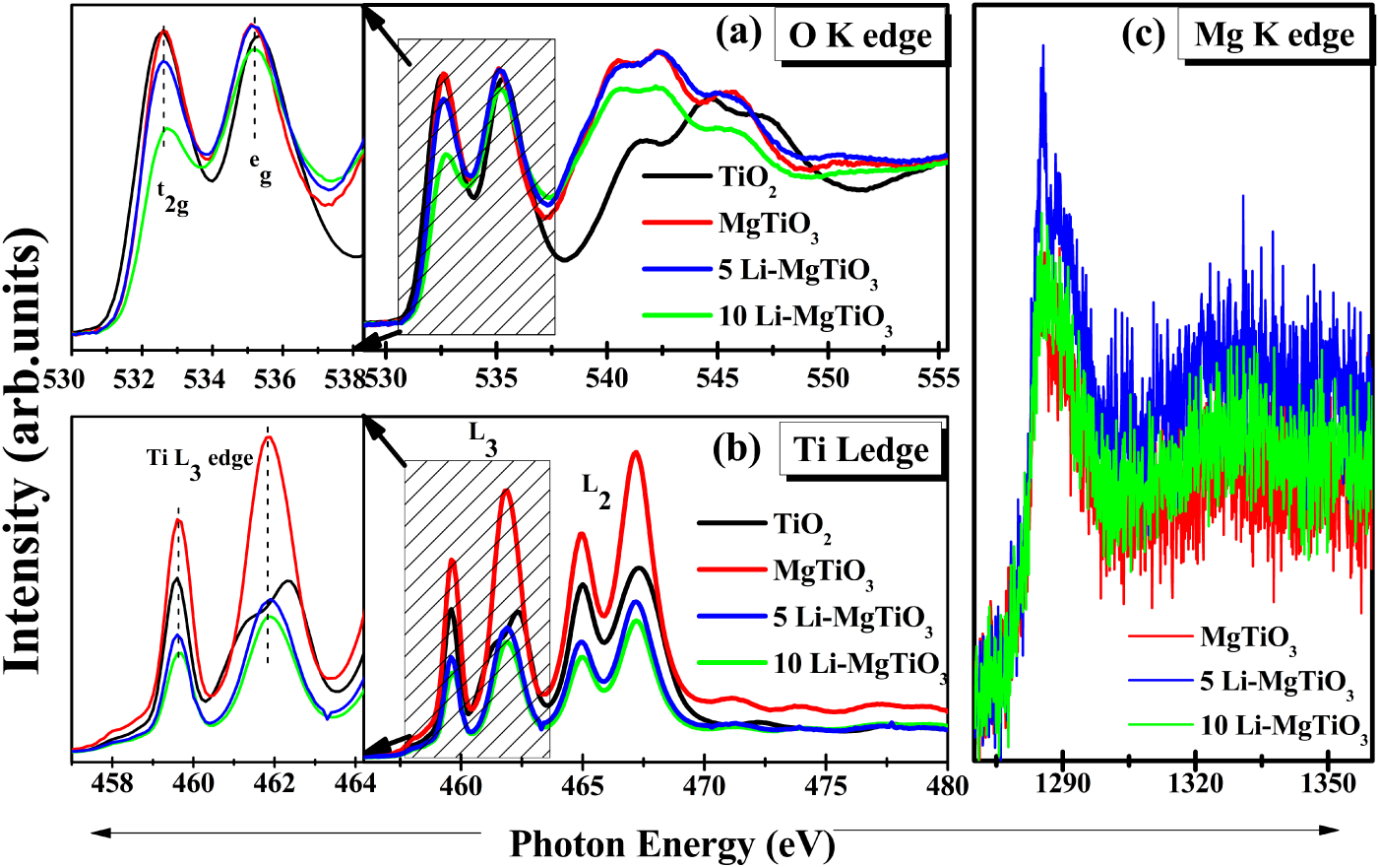
XANES spectra of (a) O K-edge, (b) Ti L-edge, and (c) Mg K-edge. The left panels of the O K-edge and Ti L-edge show magnified views of significant changes therein upon varying Li doping concentrations.

However, no significant peak position shift is observed in the O K-edge XANES, which may confirm the Ti^3+^ ion formation in the Li-doped samples.

Therefore, Li-doping-induced Ti–O linkage perturbation and oxygen ion vacancy formation are likely the causes of the decreased peak intensity of the O K-edge XANES. The XRD results of Li-doped MgTiO_3_ samples have suggested a decrease in unit cell volume, which conveys that Li atoms were randomly distributed in the MgTiO_3_ lattice at grain boundaries or interstitial sites, causing weak Ti–O networking and oxygen vacancies. The other high-energy spectral features in the O K-edge XANES appear due to hybridization between O 2p and metal (n + 1) sp orbitals.^50^ The decrease in the intensity of the spectral feature (especially in the 10Li-MgTiO_3_ sample) indicates significant oxygen ion vacancy formation and distortion in the Ti–O networks. The O K-edge spectral features of the reference TiO_2_ are distinct from those of MgTiO_3_-based samples. This again signifies a different coordination chemistry in MgTiO_3_ and because of the diverse crystal structures of TiO_2_ (tetragonal) and MgTiO_3_ (trigonal).

Figure 3b shows the Ti L_3,2_ edge XANES spectra of reference TiO_2_, pure, and Li-doped MgTiO_3_ samples. Similar to the O K-edge XANES, the Ti L-edge XANES is sensitive to the coordination chemistry changes around the Ti atom in Ti-based compounds.^50–52^ The Ti L-edge XANES spectral features arise from the excitation of core 2p electrons to the unoccupied 3d states (i.e., Ti 2p^6^3d^n^ (initial state) → Ti 2p^5^3d^n+1^ (final state) transitions). However, the strong spin–orbit coupling of the 2p core level splits it into two distinct edges, namely, L_3_ and L_2_ edges. The Ti *L*_3_-edge peak (at lower energy; ∼457.3 to 463.6 eV) arises due to Ti 2p_3/2_ → Ti 3d_5/2_ transitions, and the Ti *L*_2_-edge features (at higher energy; ∼463.6 to 468.0 eV) correspond to Ti 2p_1/2_ → Ti 3d_3/2_ transitions.^53^ Further, the L_3_ and/or L_2_ edges exhibit a doublet-like splitting due to symmetry considerations and the ionic interactions between the oxygen ligand and the Ti atoms.^46^ In the case of MgTiO_3_, the Ti atoms are surrounded by six O atoms; thus, the negative charge of O creates an electrostatic field that interacts with Ti 3d orbitals/electrons. The $d_{x^{2}-y^{2}}\;\mathrm{and}d_{z^{2}}$ orbitals (which form e_g_, states) point directly to the O ligand, experience strong interaction, and rise in energy. On the other hand, the *d*_*xy*_, *d*_*yz*_, and *d*_*zx*_ orbitals (set of t_2g_ states) are away from the vicinity of O ligands and, thus, are lower in energy.^54^ The L_3_ and L_2_ features are nearly replicas of each other. Therefore, we have examined the Ti L_3_ edge feature and presented it in the left panel of Figure 3b. The *L*_3_-edge spectra of MgTiO_3_, 5Li-MgTiO_3_, and 10Li-MgTiO_3_ show two sharp peaks, which resemble the energy position of the features of reference TiO_2_ (i.e., Ti^4+^ ions under octahedral coordination) and, thus, convey the Ti^4+^ ions with octahedral coordination chemistry. The edge energy position of Ti L_3_ or L_2_ features remains the same under different Li doping concentrations, conveying the unaltered oxidation state of Ti^4+^ ions. However, it is noticeable that the peak intensity of L_3_-edge features (both t_2g_ and e_g_) diminishes with increasing Li doping concentrations in MgTiO_3_, since the Ti L-edge peak intensity is directly proportional to the number of unoccupied 3d states.

Therefore, decreased peak intensity correlates with the occupation of 3d states. As discussed above, Li^+^ doping creates a charge and size imbalance in the host lattice. To maintain overall charge neutrality, the host lattice compensates by forming positively charged defects. The most common defect is an oxygen vacancy $\left(V_{O}^{••}\right)$ . Therefore, the formation of oxygen vacancies initially disturbs the coordination environment and Ti–O–Ti networks, which may weaken the Ti 2p → Ti 3d transitions and lead to diminished intensity of L-edge features.

Figure 3c shows the Mg K-edge XANES spectra of pure and Li-doped MgTiO_3_ samples. It is noticeable that the Mg K-edge spectral features are less resolved and, importantly, do not show a distinct difference in the edge energy position and spectral features with Li doping. The less resolved spectral features in the Mg K-edge XANES data are due to the inherently low photon flux from the soft X-ray beamline facilities (in the energy range ∼1.3 keV), combined with strong absorption losses from beamline optics and residual gas paths. Moreover, the weak fluorescence yield of Mg further degrades the signal-to-noise ratio. However, no observable changes in the white line position (∼1290 eV) convey the existence of Mg^2+^ ions in all the samples. Though the XRD results have shown a minor phase of Mg_2_TiO_4_ in the Li-doped samples, the oxidation state of Mg and Ti in this phase is the same as that of MgTiO_3_ (i.e., Mg^2+^ and Ti^4+^). Therefore, variation in the energy position (related to the different valence state) is not seen either in the Ti L-edge or Mg K-edge XANES (under the given resolution of the beamline). Moreover, the MgTiO_3_ phase dominates in each sample. Therefore, no significant features of the Mg_2_TiO_4_-related phase are observed in the XANES investigations.

### Electrochemical Investigations under the Three-Electrode Configuration

Figure 4a–c shows the cyclic voltammetry graph of the (a) MgTiO_3_, (b) 5Li-MgTiO_3_, and (c) 10Li-MgTiO_3_ samples within the potential window range of +0.1 V to +0.7 V. The measurements were performed with varying scan rates between 5 mV/s and 100 mV/s. A conventional three-electrode configuration was used to measure the electrode performance in a 1 M KOH electrolyte solution. Ag/AgCl and Pt wire were used as the reference electrode and counter electrode, respectively. It is noticeable that the current response of the (a) MgTiO_3_, (b) 5Li-MgTiO_3_ and (c) 10Li-MgTiO_3_ electrodes enhanced as scan rates increased, which demonstrates the capacitive behavior of these electrodes. The CV characteristics of the electrodes display oxidation and reduction peaks, suggesting Faradaic reactions mediated by the pseudocapacitive nature of the MgTiO_3_ compound. During the charging and discharging of MgTiO_3_ in an aqueous electrolyte solution, the titanium ions undergo reversible redox reactions via changing the Ti^4+^ into Ti^3+^ ions and vice versa: *Ti* ^4+^ + *e* ^–^ ↔ *Ti* ^3+^.^33^ The redox transitions are typically localized at the Ti–O_6_ octahedral sites within the MgTiO_3_ lattice. The Mg^2+^ ions are generally electrochemically inert under the charging and discharging conditions. However, Mg^2+^ ions play a vital role in stabilizing the crystal structure and offering high cyclic efficiency. It is noticeable that the area under the CV curves increases as a function of scan rate. This is due to the increased current during the Faradaic reactions. The CV curves of all three electrodes show oxidation and reduction peaks at 0.47 and 0.33 V, respectively, at the scan rate of 5 mV/s. As the scan rate increases from 5 mV/s to 100 mV/s, it has been observed that there is a slight shifting of oxidation and reduction peaks during the cycle. This is because, at lower scan rates, the Ti ions have enough time for ionic diffusion and require more potential to overcome the resistance in redox, leading to narrow peak separation. However, at higher scan rates, electron transfer and Ti redox cannot keep up with the rapid potential change, resulting in the shifting of oxidation/reduction peaks, which increases the peak separation.

**Figure 4:**
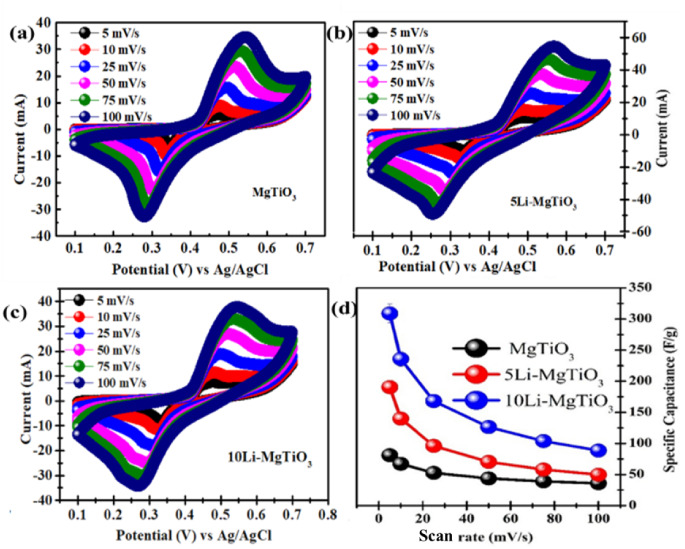
Cyclic voltammetry graphs of (a–c) pure and Li-doped samples; (d) specific capacitance of electrodes as a function of scan rate (5 mV/s to 100 mV/s).

It is also noticeable that the incorporation of Li leads to an increase in the current and the area under the CV curves, which is nearly 1.5 times higher for the 10Li-MgTiO_3_ sample compared to the bare MgTiO_3_ sample. The distinct oxidation/reduction peaks of Li are not visible, which might be due to the overshadowing effect of Ti-related oxidation/reduction features or the fast intercalation of Li. The plausible mechanism of the participation of both (Ti and Li ions) in the cyclic voltammetry involves Ti-based oxidation/reduction and Li migration. At the time of anodic (oxidation) potential, the Ti^3+^ ions oxidize to Ti^4+^, and the Li^+^ ions may move to the electrolyte via the reaction; *Ti* ^3+^ + *Li* ^+^ → *Ti* ^4+^ + *e* ^–^ + (*released to electrolyte*). Likewise, at the time of cathodic (reduction) potential, the Ti^4+^ ions are reduced to Ti^3+^, and the Li ions from the electrolyte intercalate into the Mg_1–*x*_Li_*x*_TiO_3_ structure to maintain the charge balance through the chemical reaction; *Ti* ^4+^ + *e* ^+^ + Li^+^ (*from electrolyte*) → *Ti* ^3+^+ *Li* ^+^ (*intercalate*). Thus, with Li doping in MgTiO_3_ electrodes, Li^+^ ions facilitate the redox mechanism by supporting the reversible Ti^4+^ /Ti^3+^ transitions through an intercalation/deintercalation process. The doping-induced lattice distortion and local charge imbalance enhance the availability of redox-active sites and improve ionic and electronic conductivity, leading to superior pseudocapacitive performance. Specific capacitance (C_sp_) is evaluated from the CV curves using the relation,^55^ $C_{sp}=\frac{\int I\left(V\right)dV}{mK\left(V_{2}-V_{1}\right)}$ . In this relation, the difference between two voltages is represented by *(V*_2_ *– V*_1_ *), m* is the applied mass of the material, and the scan rate is represented by *K*. The integral component is expressed by the area of the volumetric curve. C_sp_ of MgTiO_3_, 5Li-MgTiO_3_, and 10Li-MgTiO_3_ samples are 81.0 F/g, 190.5 F/g, and 309.0 F/g, respectively (at a scan rate of 5 mV/s), and these values are presented in Figure 4d. As the scan rates increase, the value of C_sp_ decreases because, at higher scan rates, the ions of the electrolyte have a shorter duration of time to enter deeply into the pores of the electrode material. As a consequence, fewer ions are involved in the charge storage process, which leads to a lower capacitance of the electrode. It is customary to note that the specific capacitance of the electrode is also influenced by the conductivity of the phase present in the compound. It is known that the conductivity of the Mg_2_TiO_4_ phase is a bit higher than that of the MgTiO_3_ phase.^56^ Therefore, in the Li-doped samples, the existence of Mg_2_TiO_4_ phase is expected to contribute to better conductivity of the electrode and assist in achieving higher specific capacitance.

To verify the charge storage mechanism in different samples, we have plotted log­(*i*; current) versus log­(*v*; scan rate) plots to estimate the parameter *b* of the power law of current and scan rates; *i* = *av* ^*b*^, which gives log­(*i*) = log­(*a*) + *b*log­(*v*), where *a* and *b* are adjustable parameters and, quantitatively, offer the diffusion and adsorption/desorption (i.e., surface) controlled capacitive nature of the samples.^47^ The 0.5 value of the *b* parameter signifies the proportionality of the current to the square root of the scan rate, and the charge storage mechanism has a diffusion-controlled battery-type nature. On the other hand, *b* = 1 indicates that the current is linearly dependent on the scan rate and the charge storage mechanism is capacitive (i.e., surface-dominating) in nature. Figure 5a– shows the log­(i) vs log­(v) plots for (a) MgTiO_3_, (b) 5Li-MgTiO_3_, and (c) 10Li-MgTiO_3_ samples, respectively. The values of coefficient *b* are mentioned in each panel of Figure 4a–c. The diffusion and surface-controlled charge storage contribution is presented in Figure 5d–f for the respective samples. It is noticeable that the surface contribution to the charge storage is significantly decreased from pure MgTiO_3_ to the 10Li-MgTiO_3_ sample (33% to 12% at a scan rate of 100 mV/s). This further supports the mechanism of Ti^4+^/Ti^3+^-related redox reactions and Li intercalation-based charge storage as discussed above.

**Figure 5:**
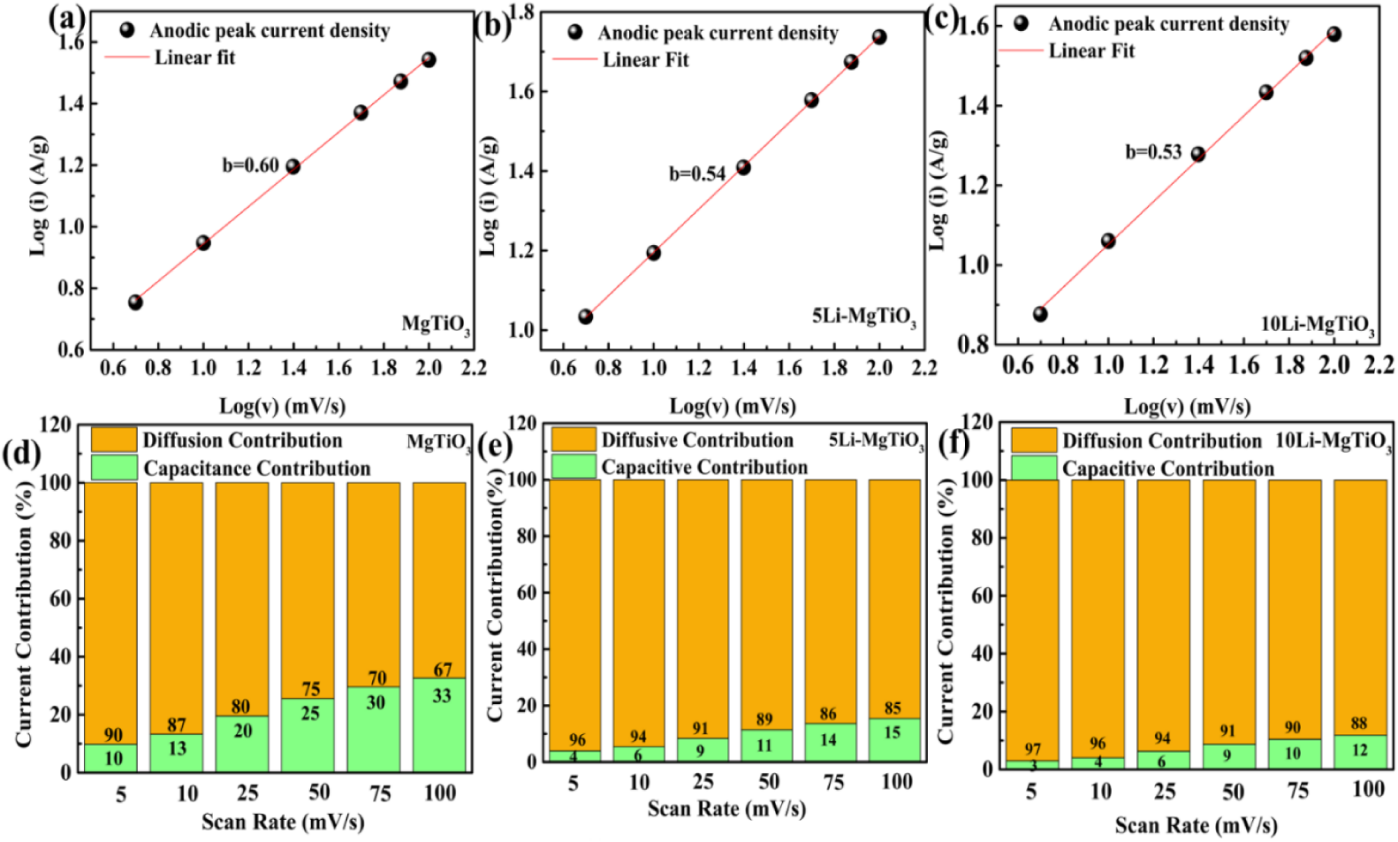
Log­(i) vs Log­(v) curves of (a) MgTiO_3_, (b) 5Li-MgTiO_3_, and (c) 10Li-MgTiO_3_ samples; and (d–f) capacitive and diffusion contribution graphs.

GCD curves are essential for analyzing the electrochemical behavior of energy storage devices. During the charging process, the accumulated ions at the electrode–electrolyte interface facilitate the transfer of electrons from the external circuit to the electrode. Conversely, during discharge, the ions return to the electrolyte by releasing the stored energy. Galvanostatic control ensures a steady current flow and helps to quantify supercapacitor energy delivery and storage capacities. Figure 6a–c illustrates the charge–discharge curves of MgTiO_3_, 5Li-MgTiO_3_, and 10Li-MgTiO_3_ samples, with a potential window ranging from +0.1 V to +0.55 V. The smaller potential window (in GCD, compared to CV data) may be due to the oxygen evolution reaction occurring in most cases of electrochemical characterization of the supercapacitor electrodes. The charge–discharge curve’s shape is nonlinear and conveys the pseudocapacitive behavior of the samples. The discharge time of Li-doped samples is better than that of the bare MgTiO_3_ sample and conveys better supercapacitor characteristics. The specific capacitance is also evaluated using the formula $C_{sp}=\frac{I\Delta t}{m\Delta V}$ from GCD curves. The estimated specific capacitance for MgTiO_3_, 5Li-MgTiO_3_, and 10Li-MgTiO_3_ samples is 55.9 F/g, 136.6 F/g, and 260.7 F/g, respectively, as shown in Figure 6d. The specific capacitance evaluated from GCD and CV measurements is comparable. Figure 7 presents the fitted electrochemical impedance spectroscopy (EIS) data for (a) MgTiO_3_, (b) 5Li-MgTiO_3_, and (c) 10Li-MgTiO_3_ samples. Three key parameters are essential in characterizing the impedance behavior: (i) series resistance (R_s)_, (ii) charge transfer resistance (R_ct_), and (iii) Warburg impedance (W_s_). The series resistance (R_s_) encompasses contributions from the intrinsic resistance of the electrode material, the ionic resistance of the electrolyte, and the interfacial resistance at the electrode/current collector junction. It is determined from the high-frequency intercept of the semicircle on the real (*x*-axis) of the Nyquist plot. The charge transfer resistance (R_ct_) reflects the resistance to charge transfer at the electrode/electrolyte interface and is represented by the diameter of the semicircle. Lower R_ct_ values indicate enhanced charge transfer kinetics at the interface. The Warburg impedance (W_s_) accounts for ion diffusion limitations at low frequencies (<0.1 Hz) and is indicative of the frequency-dependent ion transport within the electrolyte. It is mathematically expressed as W = A/(jω)^0.5^ where A is the Warburg coefficient, and ω denotes the angular frequency. In the present study, R_s_, R_ct_, and W_s_ were extracted by fitting the EIS data using the Randles equivalent circuit. The corresponding equivalent circuit is provided in the inset of Figure 7, and the fitted parameter values are provided in the table of Figure 7d. Notably, the Li-doped MgTiO_3_ samples show lower Rs, R_ct_, and W_s_ values compared with bare MgTiO_3_, indicating improved charge transport characteristics. A slight increase in R_ct_ value is observed in the 10Li-MgTiO_3_ sample compared to bare MgTiO_3_ and 5Li-MgTiO_3_. The increase in R_ct_ value at higher Li^+^ substitution indicates excessive defect formation, leading to local lattice distortion at the electrode surface. These defects act as charge-trapping centers at the electrode–electrolyte interface, which hinders electron/ion exchange. Therefore, interfacial charge transfer becomes less efficient, resulting in an increase in resistance value, whereas optimal Li^+^ substitution in 5Li-MgTiO_3_ improves conductivity and surface activity without hindering the interface.

**Figure 6:**
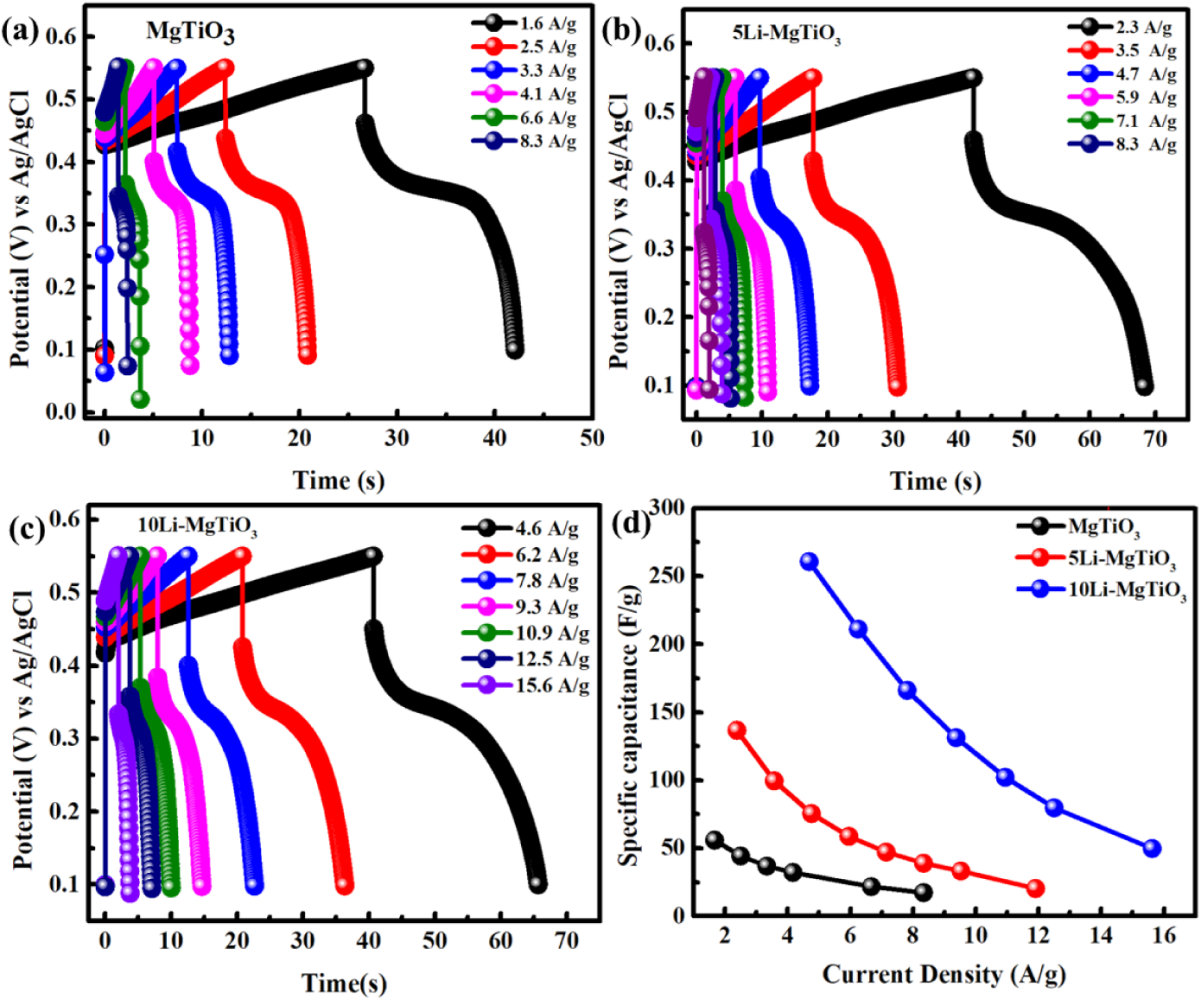
Galvanostatic charge–discharge curves of (a–c) MgTiO_3_ and Li-doped MgTiO_3_ samples; (d) specific capacitance of electrodes as a function of current density.

**Figure 7:**
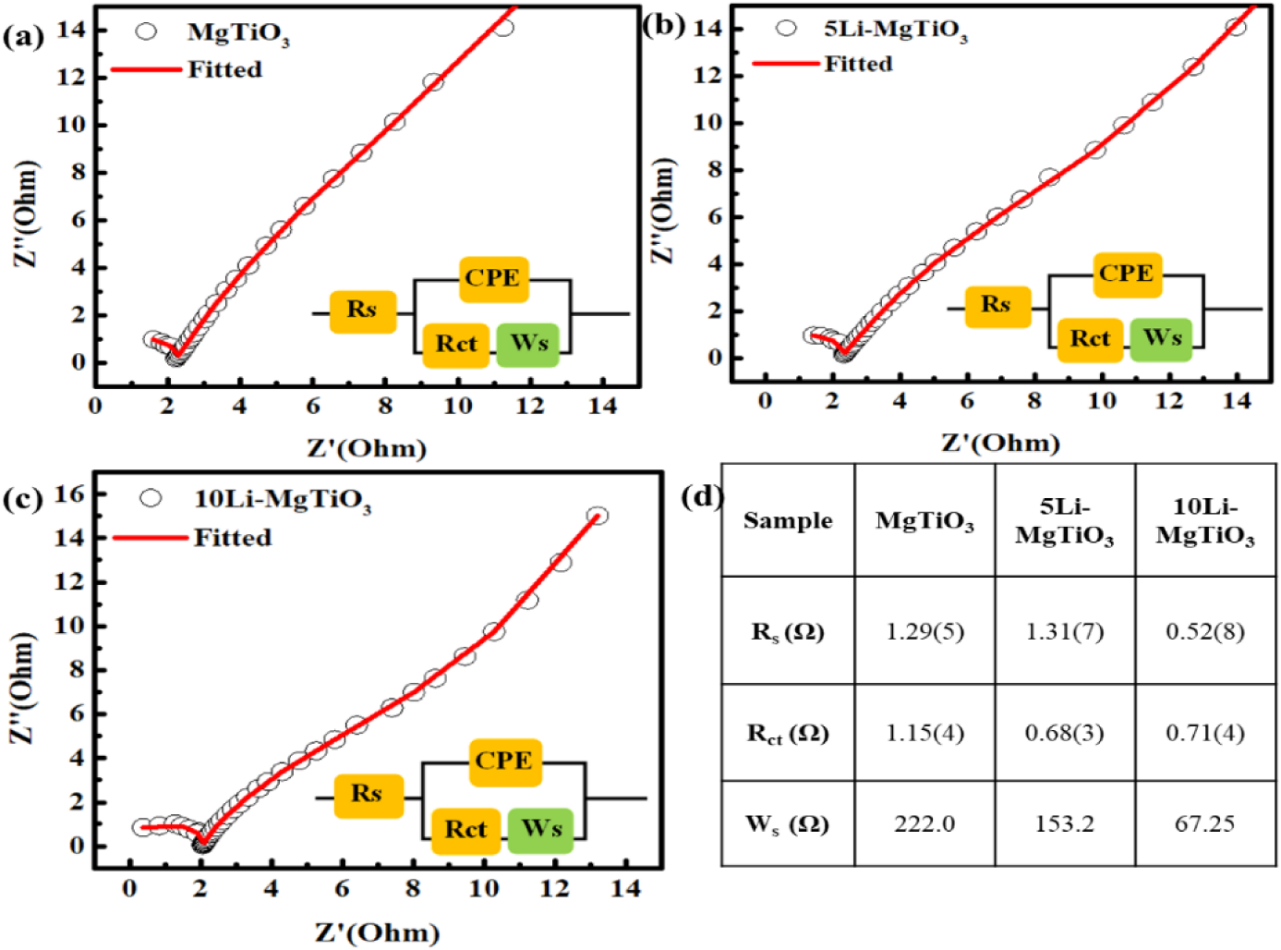
(a–c) Nyquist plots of the same set of samples (inset shows the equivalent circuits); and (d) fitted parameters (R_s_, R_ct_, and W_s_) of these samples.

### Electrochemical Investigations for the Swagelok Device under the Two-Electrode Configuration

By considering the superior electrochemical traits of the 10Li-MgTiO_3_ sample, a symmetric supercapacitor device (Swagelok cell) has been prepared. A schematic of the Swagelok cell device is presented in Scheme S3 (ESI file). Figure 8a shows the CV curves of the device at varying scan rates ranging from 5 mV/s to 100 mV/s in the wide potential window of −2 to 2 V. The wide potential window and oxidation/reduction peaks are attributed to the strong redox behavior of Ti. Figure 8b shows the GCD investigations of a two-electrode Swagelok cell. The GCD measurements are collected for different current densities and a potential window between −2 V and +2 V. The asymmetric charging and discharging characteristics are convincing evidence of the pseudocapacitive nature of the two-electrode-based device. Figure 8 c shows the cyclic retention of the two-electrode device for 1500 cycles, which is >80%. The inset of Figure 8c shows the LED glow using the two-electrode-based symmetric supercapacitor device prepared using the 10Li-MgTiO_3_ sample. Figure 8d shows the estimated energy density and power density of the device. The maximum energy density of ∼46 Wh/kg is observed at a power density of ∼5000 W/kg (at a current density of 2 A/g). The comparison of CV curves before and after long-term cycling is presented in Figure S2 (ESI file). It shows an unchanged shape and a slight drop in current response, which signifies the stability of the device. A brief comparison of the energy density/power density of the previously reported supercapacitor devices has been summarized in Table S3 (ESI file). The 10Li-MgTiO_3_-based device, prepared in this study, displays comparable cycling stability and competitive energy density, attributed to its modified electronic structure and pseudocapacitive redox activity linked to Ti species and Li-induced defect modulation.

**Figure 8:**
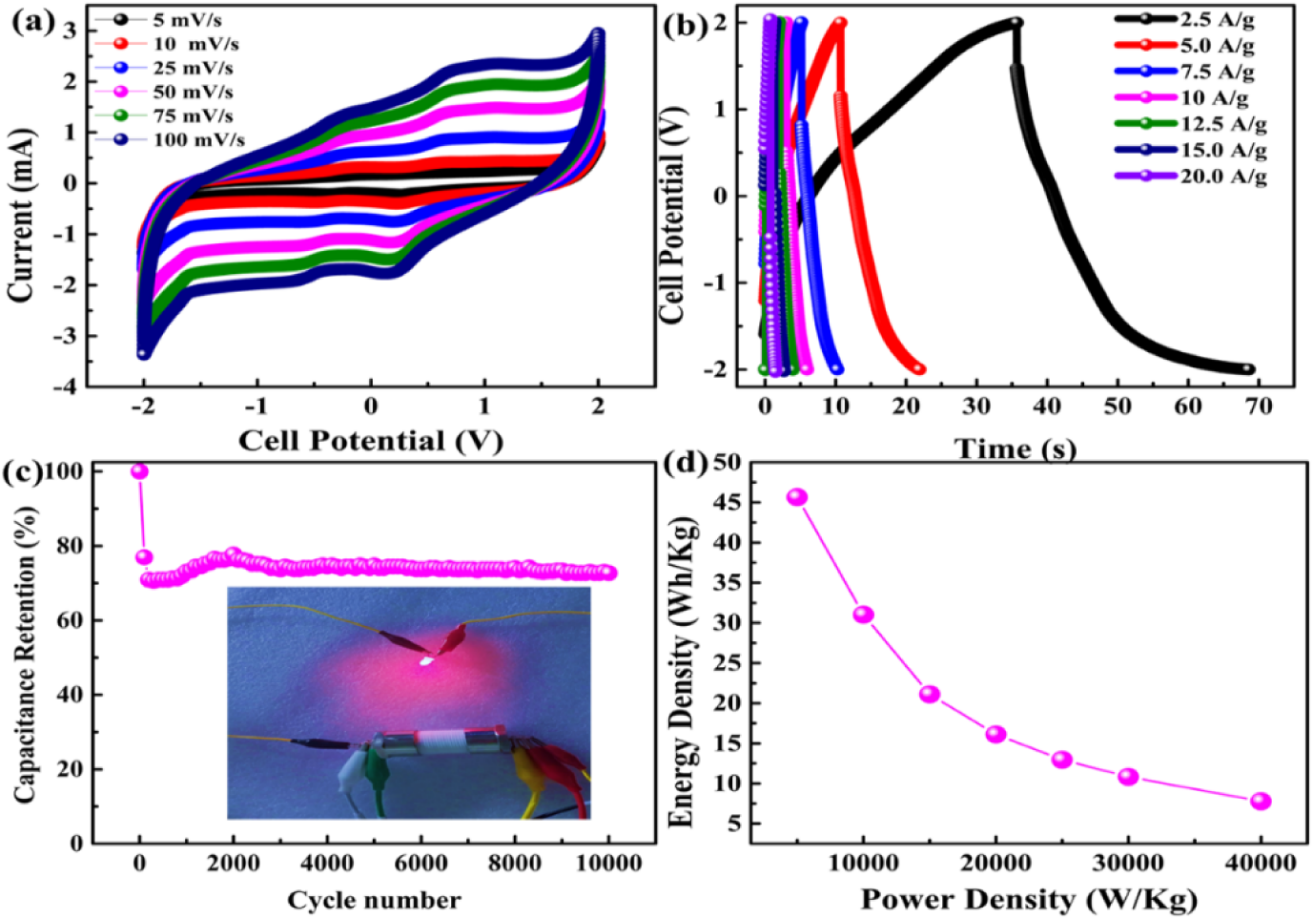
Two-electrode-based testing of the Swagelok cell prepared with the 10Li-MgTiO_3_ sample. (a) CV curves at different scan rates, (b) GCD curves at different current densities, (c) % capacitance retention as a function of the number of cycles (inset shows the glow of an LED under the two-electrode testing); and (d) power density vs energy density.

## Conclusion

In this work, the interdependence of crystal structure, electronic structure, and electrochemical energy storage behavior of Li-doped MgTiO_3_ ceramics synthesized via a solid-state reaction route has been systematically elucidated. Structural analysis confirms MgTiO_3_ as the predominant phase, with the emergence of a minor Mg_2_TiO_4_ secondary phase upon Li incorporation, while maintaining a compact morphology composed of relatively large particles. X-ray absorption spectroscopy establishes the preservation of Ti^4+^ and Mg^2+^ oxidation states across all compositions, indicating that Li doping does not induce cationic redox activity but instead perturbs the local electronic environment. Electronic structure modifications induced by Li doping are clearly reflected in XANES measurements, where a reduction in the t_2g_ and e_g_, peak intensities at the O K-edge and Ti L-edge signifies an increased occupied density of states near the Fermi level. These electronic perturbations, coupled with lattice distortion and defect formation associated with Li incorporation and secondary phase evolution, facilitate enhanced charge-transfer kinetics and ion diffusion pathways. Consequently, the electrochemical response transitions toward a synergistic surface-controlled and diffusion-controlled charge storage mechanism, resulting in a pronounced enhancement in specific capacitance from 81.0 F/g for pristine MgTiO_3_ to 190.5 F/g and 309.0 F/g for 5Li-MgTiO_3_ and 10Li-MgTiO_3_, respectively, at a scan rate of 5 mV/s. Furthermore, a symmetric supercapacitor assembled in a Swagelok configuration demonstrates a wide operational voltage window (−2 to +2 V), delivering an energy density of ∼46 Wh/kg at a power density of ∼5000 W/kg, along with excellent cycling stability exceeding ∼71% capacitance retention after 10,000 cycles. The ability to power a red LED further validates its practical applicability. Overall, this study presents that Li-induced electronic structure modulation, rather than changes in formal oxidation states, plays a decisive role in governing the electrochemical performance of MgTiO_3_, positioning Li-doped MgTiO_3_ ceramics as promising electrode materials for advanced energy storage systems.

## Supplementary Material



## Notes

Schematic diagram of synthesis method, preparation of electrode, fabrication of symmetric supercapacitor, and comparison of CV results of the device after 10,000 cycles (PDF)

## Abbreviations

XRD: X-ray diffraction
SEM: scanning electron microscope
EDLC: electrochemical double-layer capacitor
XANES: X-ray absorption near-edge structure
CV: cyclic voltammetry
GCD: galvanostatic charge–discharge
EIS: electrochemical impedance spectroscopy
