# Supplementary material for: Li-Doping-Induced Structural and Electronic Structure Modulation in MgTiO3 for an Electrochemical Energy Storage Supercapacitor Device
Source: ACS Omega. 2026 Jul 1;11(27):39865–76. doi: 10.1021/acsomega.6c00638 (PMC13382714; doi:10.1021/acsomega.6c00638)
Supplement: Supplementary file 1 [file ao6c00638_si_001.pdf]

Electronic Supplementary Information File

Li doping induced structural and electronic structure modulation in $MgTiO_3$ for an electrochemical energy storage supercapacitor device

Priyanka[1], Aditya Sharma[2*], Bhavi Agrawal[2], Mayora Varshney[3], Shalendra Kumar[2], Hyun Joon Shin[4], Keun Hwa Chae[5], Jitendra Pal Singh[1], Jai Parkash[1**]

[1]Department of Sciences (Physics), Manav Rachna University, Faridabad, 121004, Haryana, India.

[2]Department of Physics, University of Petroleum and Energy Studies, Dehradun, Uttarakhand, 248007, India.

[3]School of Applied & Life Sciences, UIT, & Division of Research & Innovation, Uttaranchal University, Dehradun, Uttarakhand 248007, India.

[4]Department of Physics, Chung Buk National University, Cheongju, 28644, South Korea.

[5]Advanced Analysis & Data Centre, Korea Institute of Science and Technology, Seoul, 02792, South Korea

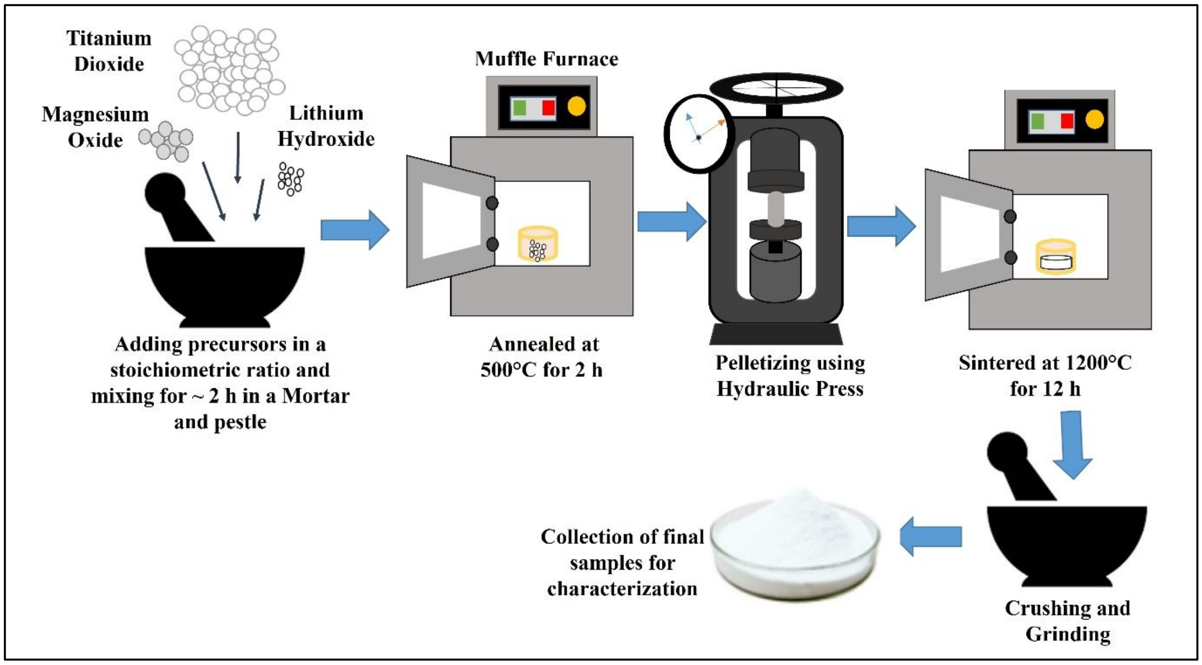

Scheme S1. Schematic for the synthesis of pure and Li-doped $MgTiO_3$ samples.

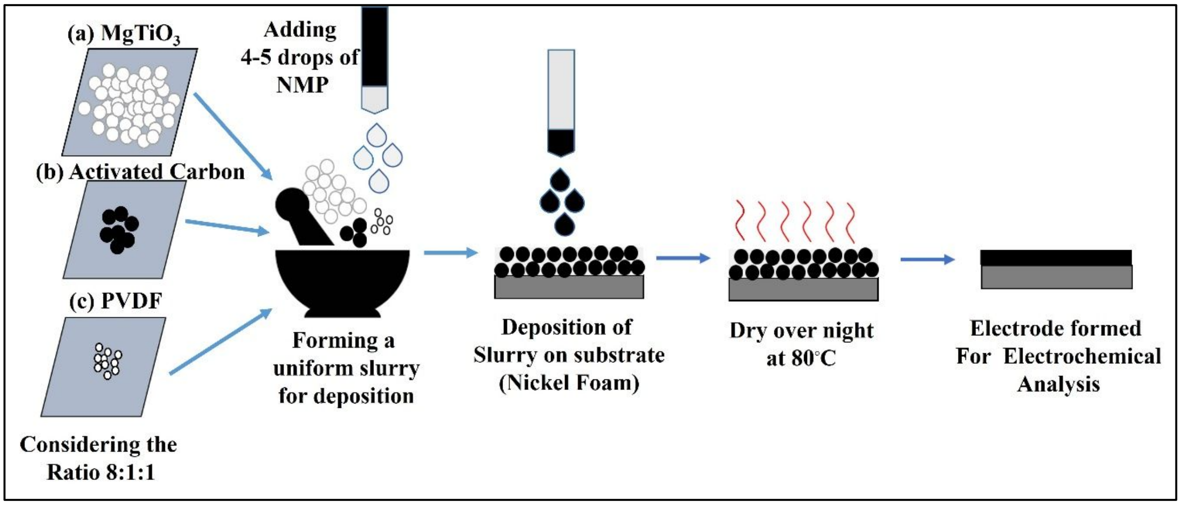

Scheme S2. Schematic of drop-cast method for the electrode's fabrication.

Table S1. Refined and reliability parameters of $MgTiO_3$, 5Li-$MgTiO_3$ and 10Li-$MgTiO_3$. Values in parentheses present error therein.

**Sample Name**	**$MgTiO_3$ Lattice parameters**			**Mg_2TiO_4 Lattice parameters**		**Rp**	**Rwp**	**χ^2**
	**a=b (A^0)**	**c (A^0)**	**Volume of unit cell $(A^0)^3$**	**a=b=c (A^0)**	**Volume of unit cell $(A^0)^3$**			
$MgTiO_3$	5.05615(6)	13.90783(5)	307.915			11.9	14.8	9.04
5Li-$MgTiO_3$	5.05495(8)	13.90350(2)	307.673	8.43923(6)	601.048	11.6	13.0	9.91
10Li-$MgTiO_3$	5.05582(0)	13.90571(6)	307.827	8.43453(3)	600.044	10.6	10.7	5.68

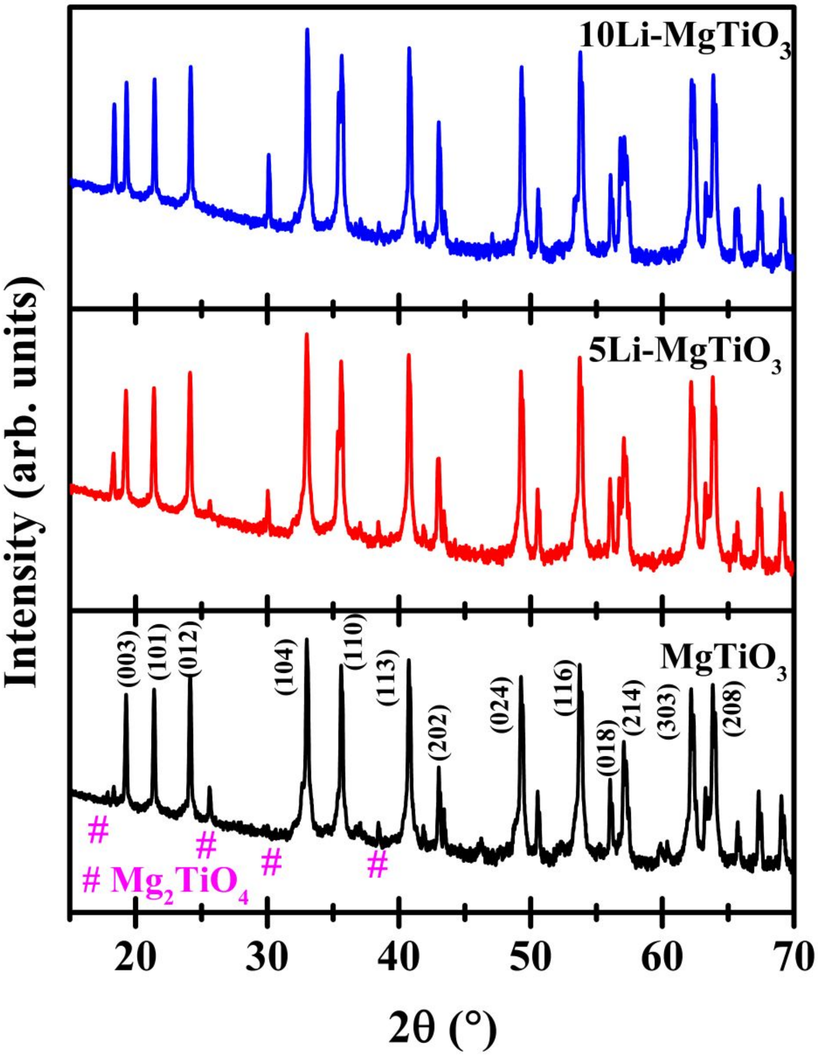

Figure S1. XRD patterns of $MgTiO_3$, 5Li-$MgTiO_3$, and10Li-$MgTiO_3$. The (#) shows XRD peaks from the $MgTi_2O_5$ phase (JCPDS Card no.06-0494). The intensity (y-axis) is taken in Log scale for better clarity of the XRD peaks from the $MgTi_2O_5$ phase.

Section S1: Electrode Configuration (three-electrode and two-electrode)

(a) 3-electrode configuration

The working electrodes were fabricated by mixing 80% of the active material (pure and Li-incorporated $MgTiO_3$), 10% polyvinylidene fluoride (PVDF) as binder, and 10% activated carbon black as conductive additive. The mixture was ground in an agate mortar pestle with gradual addition of N-methyl-2-pyyrolidone (NMP), which acts as a solvent to form a homogenous slurry. The prepared slurry was drop-cast onto nickel foam substrate (1 cm × 1 cm) of area ~ 1cm^2. The electrode was dried overnight at 80°C. Each electrode had the same dimensions and shape. The total electrode mass loading (mg/cm^2) of all three sample are given below in Table S1.

Table S2. Mass loadings in sample materials in mg/cm^2.

Sample material	Electrode mass loading before deposition (mg/cm^2)	Electrode mass loading after deposition (mg/cm^2)	Mass deposited on electrode (mg/cm^2)
$MgTiO_3$	20.0	24.8	4.8
5Li- $MgTiO_3$	21.2	24.4	3.2
10Li- $MgTiO_3$	21.7	25.9	4.2

Electrochemical measurements were performed using three-electrode cell configuration, where reference electrode was the Ag/AgCl (3M KCl), and the counter electrode was the Pt wire in 1M KOH (5.61g of KOH in 100 mL of de-ionised water) aqueous electrolyte. Cyclic voltammetry (CV) was conducted at scan rates ranging from 5 to 100 mV/s vs Ag/AgCl under potential window ranges +0.1 to +0.7 V. Galvanostatic charge-discharge (GCD) measurements were performed at various current densities ranging in between 1.6 A/g to 15.6 A/g. Electrochemical impedance spectroscopy (EIS) was carried out in the AC frequency range 1 Hz to 1MHz and the open circuit potential had amplitude of +10 mV. All measurements were conducted at room temperature (25±2°C) using Corrtest-CS2350M electrochemical system with standard three-electrode configuration.

(b) Swagelok cell testing for a symmetric supercapacitor device (2-electrode configuration)

A symmetric supercapacitor device was assembled using a Swagelok-type two-electrode cell to evaluate the practical application potential of 10Li-$MgTiO_3$. Both electrodes were prepared identically using the same composition of 80:10:10 of active material, i.e., 10Li-$MgTiO_3$, activated carbon and PVDF binder, respectively. The slurry was prepared following the procedure follows in experimental in 3-electrode configuration section. The slurry made with NMP solvent was applied on two circular graphitic paper current collectors (diameter ~14 mm) of the same dimensions to make both the electrodes of the device. The mass deposited on each electrode was ~5 mg. The separator used in supercapacitor device was Whatman grade 1 cellulose filter paper. Whatman filter paper was choosen for separator due to its high porosity and excellent wettability with aqueous electrolytes. To create a separator, a Whatman filter paper was cut with dimensions similar to those of the electrodes. This separator was soaked in 1M KOH aqueous electrolyte for 30 minutes and gently blotted to remove excess before assembling the Swagelok cell and dried air

at room temperature. The device was assembled in a swagelock cell configuration was shown in scheme S3.

The electrochemical studies of the symmetric device was evaluated with the help of CV and GCD technique. The CV curves of the device, at varying scan rates ranging from 5 mV/s to 100 mV/s, in the wide potential window of -2 V to +2 V, were collected. The wide potential window is attributed to the strong pseudocapacitive redox behavior of 10Li-$MgTiO_3$ and the overall structural stability of the material in an operating window of ~4V. GCD measurements were conducted at various current densities ranging from 0.5 A/g to 4.0 A/g. All device measurents were performed at room temperature.

(c) **Results and discussion :**

The comparison of cyclic voltammetry curves before and after long term cycling shows that the overall shape of the curve remains unchanged. Although slight drop in current response is observed after cycling. This behaviour confirms the stability of the device's electrochemical properties while indicating partial capacitance fading during prolonged operations.

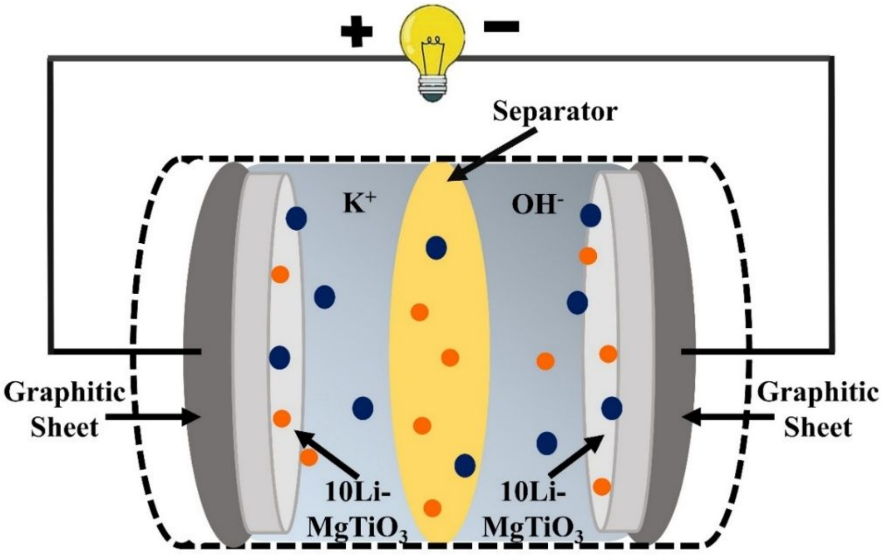

Scheme S3. Schematic of 10Li-$MgTiO_3$ based aqueous symmetric supercapacitor.

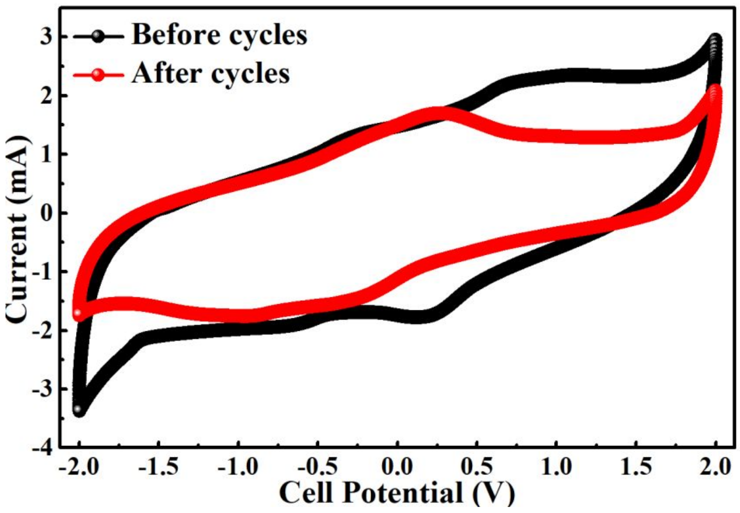

Figure S2. Cyclic viltammetry (CV) curves of the symmetric 10Li-MgTiO3 device recorded Before and after 10,000 GCD cycles with potential window of -2 to +2 V.

In order to assess the Li-doped $MgTiO_3$ symmetric device's practically. Table S2 (in supplementary information file) summarizes a comparison with recently reported metal oxide-based aqueous supercapacitors devices. In comparison to other transition metal oxides systems working in aqueous solutions, the device displays comparable cycling stability and competitive energy density. The enhanced performance can be attributed improved electronic structure, pseudocapacitive redox activity linked to Ti species and Li-induced defect modulation are responsible for the improved performance.

Table S3. Recently reported aqueous supercapacitors.

**Material**	**Electrolyte**	**Device Type**	**Capacitance (F/g)**	**Energy Density (Wh/kg)**	**Power Density (W/kg)**	**Retention**	**Reference**
ZnO/ activated carbon composite	1M KOH	Symmetric	5.8			~70% (1000 cycles)	[1]
$FeCo_2O_4$	6 M KOH	Asymmetric	88	24	-	93% (5000 cycles)	[2]

Dy_2S_3		Symmetric	26	17	520	79% (5000 cycles)	[3]
Nb//TiO_2	3 M KOH	Asymmetric	-	16.3	770	100% (5000 cycles)	[4]
α-Fe_2O_3			1.6	0.063	225	76% (1000 cycles)	[5]
**10Li-$MgTiO_3$**	**1M KOH**	**Symmetric**	**41**	**46**	**5000**	**~71% (10,000 cycles**	**This work**

Refrences :

(1) Yadav, M. S.; Singh, N.; Bobade, S. M. Zinc Oxide Nanoparticles and Activated Charcoal-Based Nanocomposite Electrode for Supercapacitor Application. *Ionics 2018 24:11* **2018**, *24* (11), 3611–3630.

(2) Tajik, S.; Dubal, D. P.; Gomez-Romero, P.; Yadegari, A.; Rashidi, A.; Nasernejad, B.; Inamuddin; Asiri, A. M. Nanostructured Mixed Transition Metal Oxides for High Performance Asymmetric Supercapacitors: Facile Synthetic Strategy. *Int. J. Hydrogen Energy* **2017**, *42* (17), 12384–12395.

(3) Bagwade, P. P.; Nikam, R. P.; Bhosale, R. P.; Khot, S. D.; Lokhande, C. D. Performance of Solid-State Symmetric Supercapacitors Based on Dy_2S_3 Electrodes. *Applied Surface Science Advances* **2023**, *18* (11), 100529.

(4) Vidyadharan, B.; Archana, P. S.; Ismail, J.; Yusoff, M. M.; Jose, R. Improved Supercapacitive Charge Storage in Electrospun Niobium Doped Titania Nanowires. *RSC Adv.* **2015**, *5* (62), 50087–50097.

(5) Khatavkar, S. N.; Sartale, S. D. Fabrication and Evaluation of Symmetric Flexible Solid State Supercapacitor Device Based on α-Fe_2O_3 Thin Films by LPD. *AIP Conf. Proc.* **2021**, *2335* (1).
